# The effect of a startle-based warning, age, sex, and secondary task on takeover actions in critical autonomous driving scenarios

**DOI:** 10.3389/fbioe.2023.1147606

**Published:** 2023-03-27

**Authors:** M. Griffith, R. Akkem, J. Maheshwari, T. Seacrist, K. B. Arbogast, V. Graci

**Affiliations:** ^1^ Center for Injury Research and Prevention, Children’s Hospital of Philadelphia, Philadelphia, PA, United States; ^2^ School of Biomedical Engineering, Science, and Health Systems, Drexel University, Philadelphia, PA, United States; ^3^ Perelman School of Medicine, University of Pennsylvania, Philadelphia, PA, United States

**Keywords:** reaction time, ASPS, distraction, out of the loop, driverless vehicles, out of position

## Abstract

**Introduction:** In highly autonomous driving scenarios, it is critical to identify strategies to accelerate reaction times since drivers may take too long to take over control of the vehicle. Previous studies reported that an Acoustic Startling Pre-Stimulus (ASPS, i.e., a loud warning preceding an action) accelerated reaction times in simple ankle flexion exercises.

**Methods:** In this study, we examined if an ASPS warning leads to shorter takeover reaction times in a sled-simulated evasive swerving maneuver. Twenty-eight participants (seven male adults, seven male teenagers, seven female adults, and seven female teenagers) were instructed to align a marker on the steering wheel with a marker on a lateral post as fast as they could as soon as the lateral sled perturbation (0.75 g) started. Four conditions were examined: with and without an ASPS (105 dB, played 250 ms before sled perturbation for 40 ms), and with and without a secondary task (i.e., texting). A catch trial (ASPS only) was used to minimize anticipation. Human kinematics were captured with an 8-camera 3D motion capture system.

**Results:** Results showed that the drivers’ hands lifted towards the steering wheel more quickly with the ASPS (169 ± 55 ms) than without (194 ± 46 ms; *p* = 0.01), and that adult drivers touched the steering wheel quicker with the ASPS (435 ± 54 ms) than without (470 ± 33 ms; *p* = 0.01). Similar findings were not observed for the teen drivers. Additionally, female drivers were found to lift their hands towards the steering wheel faster than male drivers (166 ± 58 ms vs. 199 ± 36 ms; *p* = 0.009).

**Discussion:** Our findings suggest that the ASPS may be beneficial to accelerate driver reaction times during the initiation of a correction maneuver, and that autonomous vehicle warnings may need to be tailored to the age and sex of the driver.

## 1 Introduction

As autonomous vehicles become a reality, there will be instances in which drivers need to take over control of the vehicle to perform a crash-avoidance maneuver. Previous studies have shown that longer takeover reaction times increase collision risk (Loeb et al., 2019). As vehicle autonomy increases, drivers take longer to resume control of the vehicle as they are out of the loop of continuously monitoring driving (Victor et al., 2018). Increased vehicle autonomy also allows drivers to engage in secondary tasks, which may lead to even longer takeover reaction times (Eriksson and Stanton, 2017). Previous studies found that drivers accepted a greater number of secondary tasks during automated driving than during manual driving (Wandtner et al., 2018a). Furthermore, studies found that of different secondary task modalities, a handheld visual-manual task (e.g., texting) increased takeover reaction times the most (Wandtner et al., 2018b).

To address concerns about drivers’ preparedness to react to a takeover request, a number of previous studies have evaluated how different in-vehicle warning systems affect reaction times and driver perceptions. One study on autonomous driving takeover found that combined visual-auditory warnings reduced hand on wheel time by an average of 3.9 s compared to visual warnings (Naujoks et al., 2014). Another study found that an audio warning was effective at prompting drivers to move their hands towards the steering wheel, even in the presence of a visual-manual distracting task (Morando et al., 2020). A study on forward collision warnings (FCW) during manual driving scenarios found that acoustic characteristics of the FCW, such as peak-to-total-time ratio, interburst interval, number of harmonics, frequency, and pulse duration, significantly affect the efficacy of auditory warnings (Lewis et al., 2017). However, many studies testing the efficacy of warning systems are conducted in driving simulators, in which drivers do not experience a real physical perturbation as they would in a real crash avoidance scenario. Such physical perturbations could cause a startle reflex (Sanders et al., 2015) and alter drivers’ takeover reaction time.

Previous studies on warning systems in vehicles have not accounted the startle reflex. Startle responses are neuromuscular body reactions to intense stimuli. According to Mang et al. (2012), startle evoked by a rear-end collision may contribute to whiplash injuries, but providing an acoustic warning just at the threshold of startle reactivity (105 dB) before the collision reduced the startle response caused by the collision and reduced head acceleration, potentially reducing the severity of whiplash injuries (Mang et al., 2012). An effective acoustic warning was determined to be an Acoustic Startling Pre-Stimulus (ASPS), which is defined as a 105 dB sound preceding the sled perturbation by 250 ms (Mang et al., 2012). Sutter and others found that the ASPS could also accelerate prepared, unpracticed movements such as ankle flexion exercises by an average of 96 ms in first trials and by an average of 33 ms in subsequent trials (Sutter et al., 2016). This phenomenon of a startling acoustic stimulus accelerating the onset of a prepared movement is known as the StartReact effect (Nonnekes et al., 2015; Sutter et al., 2016). Our group has previously investigated the effect of the ASPS on steering accuracy in autonomous driving takeover scenarios, and the results showed that adult drivers’ steering accuracy improved with the ASPS (Graci et al., 2021). ASPS was also found to reduce reaction time in adult drivers during takeover (Graci et al., 2019). However, the full effect of ASPS on takeover reaction times of drivers of different sexes and ages is still unclear, as these previous studies only analyzed steering accuracy or only included male subjects in the analysis of reaction times. Therefore, this study represents an extension and more complete assessment of the role of ASPS in accelerating takeover reaction times across drivers of different sexes and ages.

Startle response may vary by sex. Kofler et al. (2001) showed that startle reactivity is greater in women than men, in both probability and duration of response, which may impact the efficacy of ASPS. Moreover, sex differences have been found in driving behavior and crash injury risk. According to a report published by the National Highway Traffic Safety Administration (NHTSA), female drivers are overall at a higher fatality risk than male drivers given similar physical insults (Kahane, 2013). Additional studies have found female drivers to be at greater risk of higher severity injuries than male drivers even after controlling for variables such as age, height, and vehicle; however, trends of studying potential sex differences in crash involvement are recent and most past research focused on developing injury metrics and safety measures for mid-sized male occupants (Forman et al., 2019). Furthermore, female drivers have been shown to perceive risk in driving differently than male drivers, suggesting different behavior surrounding critical scenarios (Rhodes and Pivik, 2011). In a naturalistic study observing driving behavior of 100 drivers on the road over the course of a year, female drivers were found to brake earlier than male drivers, suggesting that female drivers show an earlier recognition of collision risk and decreased risk-taking tendencies as compared to male drivers (Montgomery et al., 2014). Yet, simple reaction-time tasks such as pressing a button have shown females to generally have slower reaction times than males (Der and Deary, 2009), suggesting a physiological difference between the sexes that may impact takeover reaction times.

Another factor that may influence ASPS efficacy and takeover reaction times is driver age, particularly between teenage and adult drivers. Previous studies have shown that startle reactivity is greater at younger ages, but did not consider teenagers in their study design (Ludewig et al., 2003). Some studies on takeover suggest that age may be a factor in takeover reaction time and success rate, showing adult drivers to react quickest and crash the least amongst teenage, adult, and senior drivers (Loeb et al., 2021), though research in this area is still limited. In a meta-analysis of 129 studies, Zhang et al. (2019) found no clear effect of age on takeover time; however, not all studies evaluate teenage drivers separately from young adult drivers. Teenage drivers have been shown to have different driving behaviors and crash risk than adult drivers. An analysis by the Insurance Institute for Highway Safety (IIHS) found that the fatal crash rate per mile driven for teenage drivers is nearly 3 times that of adult drivers (IIHS, 2020). Teenagers often display increased risk-taking behaviors, including in driving, likely due to cognitive developmental changes (Steinburg, 2010; Khurana et al., 2018). A study using NHTSA’s National Motor Vehicle Crash Causation Survey data found that recognition errors including inadequate surveillance and distraction were the leading cause of crashes among teenage drivers (Curry et al., 2011). Studies monitoring cell phone use during driving have shown teenage drivers to engage in cell phone use while driving, even at high speeds (McDonald et al., 2019). Previous studies have found that young male drivers tend to brake closer to the time to collision than other driver populations and that FCW systems, which occur early relative to the time to collision, may activate too close to the normal driving behavior of young males and produce too many false positives, which may lead to these young drivers deactivating the FCW (Montgomery et al., 2014). An ASPS takeover request warning may therefore be beneficial to this group, as it consists of a single beep activated 250 ms before the perturbation and may trigger fewer false positive nuisance alarms.

Therefore, the aim of this study is to examine if an ASPS decreases takeover reaction time when drivers are prepared to react (i.e., not engaged in a secondary task) and when drivers are not prepared to react (i.e., engaged in a secondary task), and to examine if sex and age influence the effectiveness of ASPS.

## 2 Methods

The study protocol was reviewed and approved by the Institutional Review Board of the Children’s Hospital of Philadelphia.

### 2.1 Participants

Twenty-eight participants, grouped as seven male adults, seven male teenagers, seven female adults, and seven female teenagers (anthropometric and demographic reported in Table 1) participated in the study. The sample size for each group was based on a previous study using the same methodology (Graci et al., 2019). To be included in the study, participants’ BMI had to be between the 5th and 95th percentile for the United States population according to the participant’s age and they needed to hold a valid driver’s license. Teenagers were included if they were under the age of 18 years old had driven at least 12 h in the previous 12 months. Adults were included if they were over the age of 25 years old and had at least 5 years of driving experience. The age range of the teenage group was small in order to capture relatively inexperienced drivers for this group, while only adults above 25 years old were included in order to select experienced adults. Thus, the age ranges were designed so that the teenage group resembled inexperienced drivers and the adult group resembled experienced drivers. This was done to minimize the effect of driving experience as a confounding factor in our small groups of volunteers. Participants in both age groups were verbally screened and excluded for self-report of any significant neuromuscular, connective tissue, orthopedic, cardiovascular, or neurologic conditions. Adult participants and parents/guardians of teenage participants provided written consent and teenage subjects provided their assent before participating in the study.

**TABLE 1:** Mean and standard deviation of age, height, and weight for each subject group.

	Age (years)	Height (cm)	Weight (kg)
Male adult (*n* = 7)	29.9 ± 4.3	177.9 ± 6.0	78.0 ± 12.9
Male teenager (*n* = 7)	17.5 ± 0.2	176.3 ± 7.2	67.3 ± 7.3
Female adult (*n* = 7)	26.3 ± 1.4	168.7 ± 5.7	59.7 ± 7.0
Female teenager (*n* = 7)	17.4 ± 0.3	168.3 ± 4.3	62.9 ± 11.3

### 2.2 Sled apparatus and instrumentation

Participants were seated on a vehicle driver’s seat within a driver compartment on a sled apparatus (Supplementary Material, Supplementary Figure S1) and restrained with 3-point seat belt. The driver compartment on the sled was designed to mimic a driver’s seating environment and was equipped with an adjustable driver’s seat (adjustable forward and vertical translations and seatback recline angle), a steering wheel, accelerator and brake pedals, a centre console, a three-point belt and adjustable B/C-pillar and D-ring structure (adjustable forward and vertical translations) (Holt et al., 2019). Three lightweight belt webbing load cells (6200FL-4130, Denton ATD Inc., Rochester Hills, MI) were installed on the shoulder belt and the right and left lap belt. The load cell data were sampled at 10 kHz using an onboard TDAS data acquisition system (TDAS Pro, DTS Inc., Seal Beach, CA). The driver compartment also included three onboard GoPro HERO Session four cameras oriented in the overhead perspective of the participants, in the frontal perspective of the participants, and at the participants’ feet. Video data was captured at 30 Hz.

**FIGURE 1:**
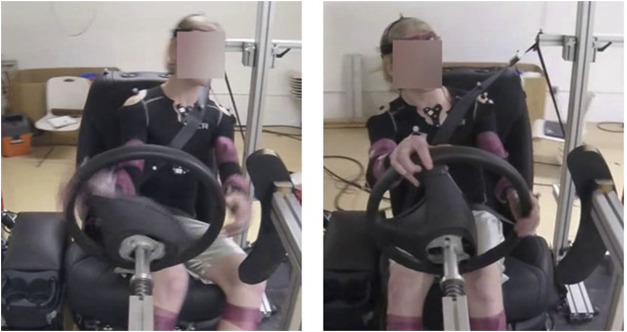
Drivers’ motion during the lateral sled perturbation, consisting of first a right swerve and motion into the belt (left image), followed by a left swerve and motion out of the belt (right image).

Participants were exposed to a low acceleration lateral sled perturbation (0.75 g) which mimicked an evasive emergency swerve (Kent et al., 2016; Holt et al., 2019). The driver compartment travelled 1.8 m laterally in 1.02 s and then reversed direction. One oscillatory movement (i.e., cycle) was provided, consisting of one right swerve (driver’s motion into the belt, Figure 1, left) followed by one left swerve (driver’s motion out of the belt, Figure 1, right). The sled acceleration was collected at 10 kHz by an accelerometer (7380a-10, Endevco, San Juan CA) mounted on the lower base of the sled frame.

### 2.3 Human subject instrumentation

Kinematic data was captured using on onboard Optitrack Prime 13 W 8-camera motion captures system (200 Hz, NaturalPoint Inc., Corvallis, OR). Calibration of the motion capture system was performed prior to each test session to determine the relative position and field of view of each camera and the global coordinate system. Participants were provided with an athletic compression shirt and a pair of athletic shorts. Once in proper attire, photo-reflective markers were placed on the participants’ head (on a tightly fitted headpiece across the temples, forehead, and head top), trunk (bilateral acromion, suprasternal notch), and upper extremities (bilateral humeral epicondyle, radial styloid process, metacarpal index). In order to minimize motion artefact and best approximate skeletal movement, the markers on the participants were placed directly on the skin by cutting holes into the provided compression shirt. For the suprasternal notch and the head, an array of four to six markers were placed on rigid structures that were then attached to the skeletal landmark, which would then be represented by a set of markers with a centre of mass in the Motive Tracker software (NaturalPoint Inc., Corvallis, OR). Other markers were placed on the top of the steering wheel, on a post placed laterally to the steering wheel, on each of the pedals, on the seat, on the seatbelt, and on the D-ring.

### 2.4 Experimental procedure

Prior to experimental trials, key anthropometric data was collected, including age, height, weight, seated height, and other torso and leg measurements.

Participants were then seated in the driver compartment on the sled and restrained with a standard 3-point seatbelt over their left shoulder. The participants’ starting position was “non-tensed” (subjects were asked to relax) with their hands in their lap. Participants were told that the task represented a highly autonomous driving scenario where they did not need to keep their hands on the steering wheel. Participants were told that in some conditions, they would be given a mobile phone; in those conditions, the participants’ starting position was “non-tensed” (subjects were asked to relax) with their hands on the phone. If participants chose to interact with the phone with only one hand, they were asked to keep the other hand on their lap. No instructions were given about their feet placement in relation to the pedals; participants were told they could use the pedals if they wanted to.

**FIGURE 2:**
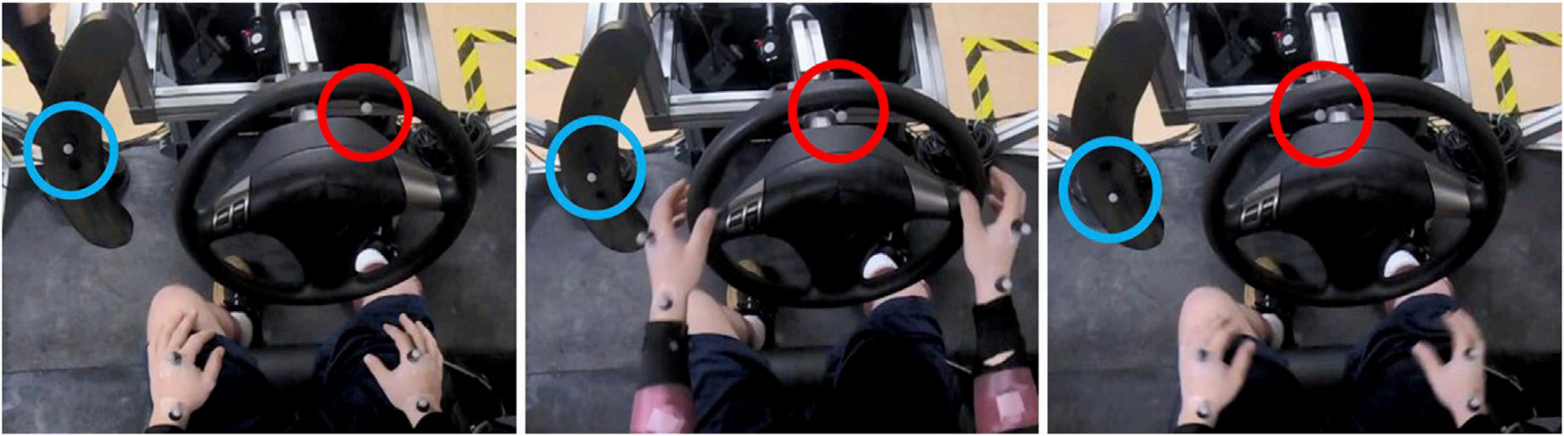
Initial position of photo-reflective markers on the steering wheel (red) and photo-reflective marker on the lateral post (blue) for each of the three positions (left, center, right images).

**FIGURE 3:**
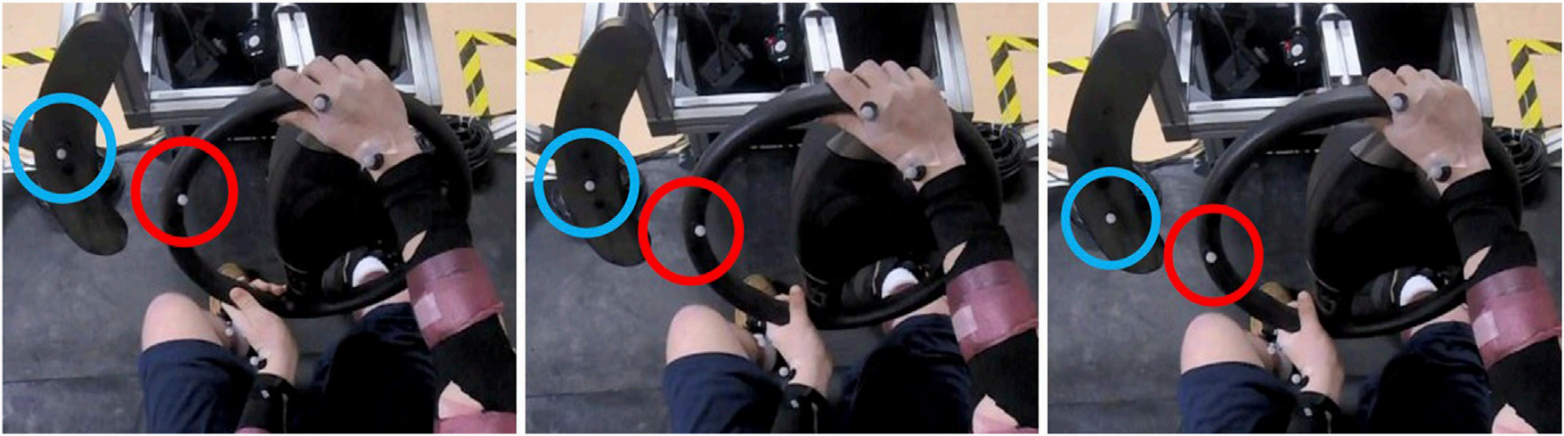
Example of alignment positions of the photo-reflective marker on the steering wheel (red) and photo-reflective marker on the lateral post (blue) for each of the three positions (left, center, right images).

The experimental task participants were instructed to perform was to align a marker on a steering wheel with a marker on a lateral post (Figures 2, 3) as quickly and accurately as they could as soon as a lateral sled perturbation began. The starting positions of the two markers were randomized between trials so that the distance between the two markers (36 cm) and angle formed by the two markers and the center of the wheel (70 deg) was always the same in the starting position, but the locations varied among three positions 4.5 cm apart (Figure 2) to minimize motor adaptation without requiring a different muscle strategy per position. Prior to testing, while the sled was stationary, participants were instructed to perform an “alignment trial” for each of the three marker positions at their comfortable speed to establish a working definition of marker alignment for each position (Figure 3).

Participants were exposed to four different testing conditions (Table 2), each repeated twice, in a randomized order. Conditions included with and without an ASPS warning, occurring at 105 dB beginning 250 ms before the sled motion and lasting for 40 ms (Mang et al., 2012), and with and without a secondary task, which consisted of typing of mobile text message. For the conditions with the texting task, participants were instructed to start typing a few seconds before the sled motion began. Participants were given text prompts requiring longer than the time for the sled motion to begin, including reflection and typing; examples included listing 10 favorite foods, listing 10 favorite spots in Philadelphia, listing 10 favorite movies, or listing 10 places they would like to visit.

**TABLE 2:** Testing conditions, repeated twice in a randomized order.

Experimental condition	Description
Sled Only	Sled perturbation only, without ASPS and without Secondary Task
Secondary Task + Sled	Sled perturbation without ASPS but with Secondary Task; sled perturbation occurred while participants typed a mobile text
ASPS + Sled	Sled perturbation with ASPS but without Secondary Task; ASPS (105 dB auditory tone lasting 40 m) was played 250 m before the sled perturbation started
Secondary Task + ASPS + Sled	Sled perturbation with ASPS and with Secondary Task; ASPS (105 dB auditory tone lasting 40 m) was played 250 m before the sled perturbation started and while participants typed a mobile text
ASPS Only (Catch trial)	ASPS (105 dB auditory tone lasting 40 m) played and no sled perturbation occurred

A fifth condition consisting of an ASPS warning only, without sled perturbation, was used as a catch trial to prevent anticipation of the sled motion. Potential anticipatory effect was also mitigated by employing a random latency time between 1 and 10 s between the experimental instruction and the sled activation, so that participants could not predict when the sled would start. A complete description of the five experimental conditions is listed in Table 2.

### 2.5 Data analysis

Kinematic data from the motion capture system was processed using Motive Tracker software (Motive 2.2.0, NaturalPoint Inc., Corvallis, OR) and then imported into custom-made Matlab (Matlab 2017b; MathWorks Inc., Natick, MA) programs to extract the relevant reaction time and kinematic outcome measures for analysis. Three reaction time measures were chosen to capture all stages of the takeover action, from orientation (e.g., motor readiness) through initialization (e.g., physical readiness) to action-execution (e.g., maneuver completed) (Cao et al., 2021) to analyze potential differences between groups and/or conditions across the entire takeover action. The specific reaction time outcome measures were: Hand-Lift-Off Time to capture the orientation stage (i.e., the time from the sled onset for drivers to lift their hand off of their lap), Hand-On-Wheel Time to capture the initialization stage (i.e., the time from the sled onset for drivers to touch the steering wheel), and Corrective Time to capture the action-execution stage (i.e., the time from the sled onset for drivers to align the steering wheel and lateral post markers). Marker alignment for the Corrective Time was defined as the closest position to their pre-defined alignment position reached by the steering wheel marker during the trial; precision of the alignment was not included in the scope of this study but has been previously analyzed by our group (Graci et al., 2021). The kinematics outcome measures were: Peak Lateral Head Displacement Into-the-Belt, Peak Lateral Head Displacement Out-of-the-Belt, Peak Lateral Trunk Displacement Into-the-Belt, and Peak Lateral Trunk Displacement Out-of-the-Belt. A Mixed Repeated Measure 4-way ANOVA was performed to understand the effect of ASPS, Age, Sex, and Secondary Task on the reaction time and kinematic outcome measures averaged over repetitions. Post-hoc tests were performed using Tukey’s HSD with level of significance set to *p* = 0.05.

Additionally, kinematic data from motion capture, verified by video analysis, were used to qualitatively describe takeover strategies, such as the use of one or two hands to touch the steering wheel. Takeover strategies were compared across testing conditions and participant groups. For trials where only one hand was used to touch the steering wheel, proportions of trials in which the first hand to lift (i.e., the hand, left or right, used to calculate Hand-Lift-Off Time) was the same as the first hand to touch the steering wheel (i.e., the hand, left or right, used to calculate Hand-On-Wheel Time) were quantified and compared across testing conditions and participant groups.

## 3 Results

**FIGURE 4:**
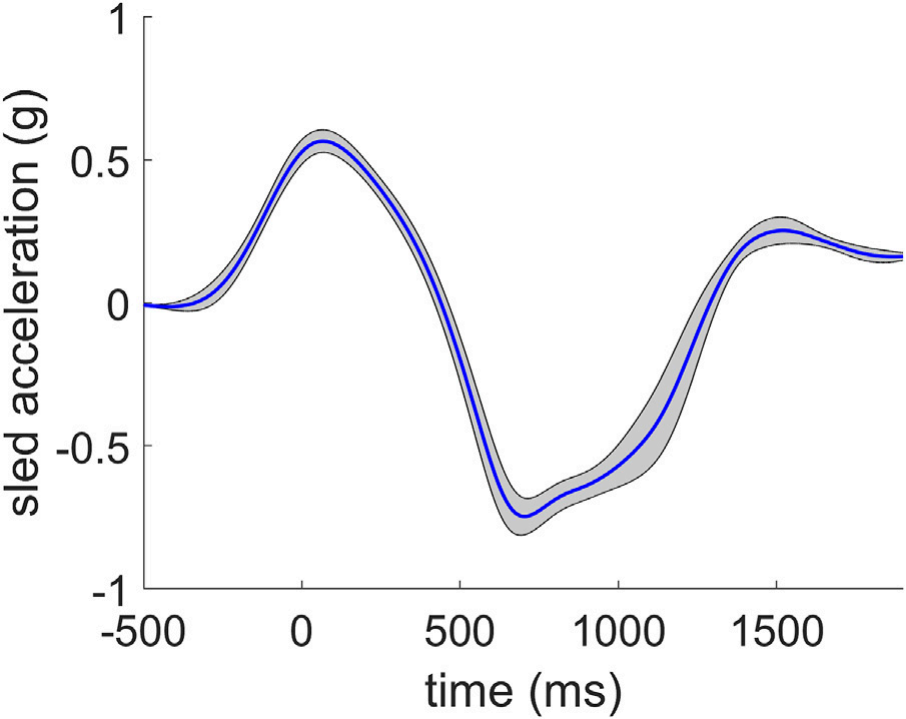
Mean and standard deviation of sled acceleration for all subjects and all trials (*n* = 224).

The mean and standard deviation of the sled acceleration during the oscillatory movement over all subjects and trials is plotted in Figure 4, showing repeatable sled motion.

Data reduction note: Hand-Lift-Off Time was extracted from 27 subjects; this measure was not extracted from one male adult subject, for whom the wrist marker data was incomplete. Data for all other dependent measures represent all 28 subjects.

### 3.1 Reaction times

#### 3.1.1 ASPS

**FIGURE 5:**
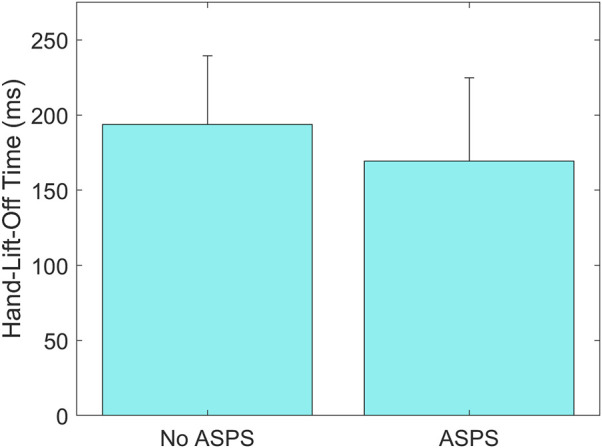
Group means ± standard deviation (SD) for Hand-Lift-Off Time without and with the ASPS.

**FIGURE 6:**
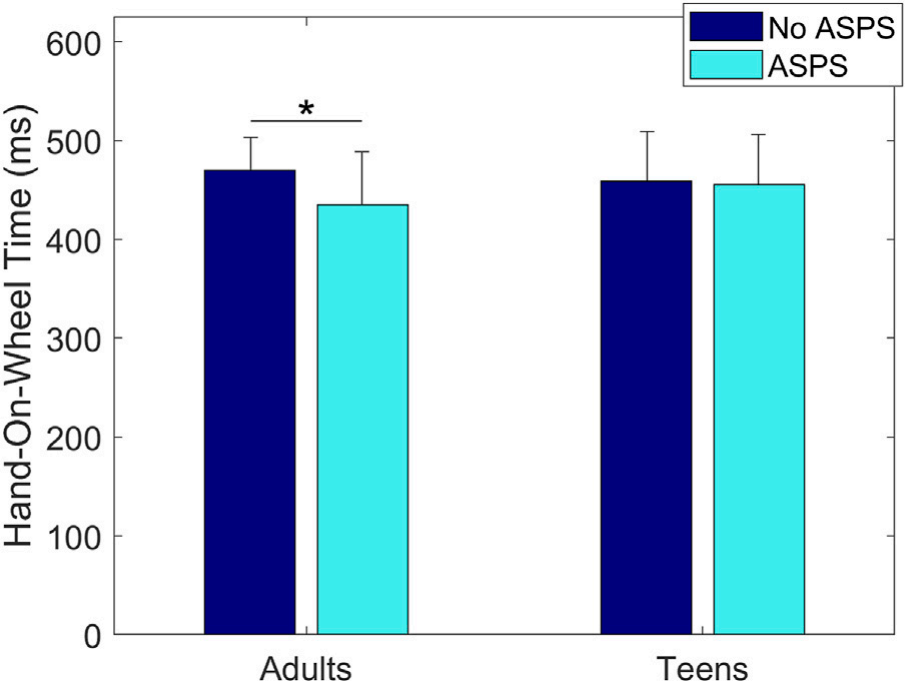
Group means ± SD for Hand-On-Wheel Time without and with the ASPS for adult drivers and teenage drivers.

**FIGURE 7:**
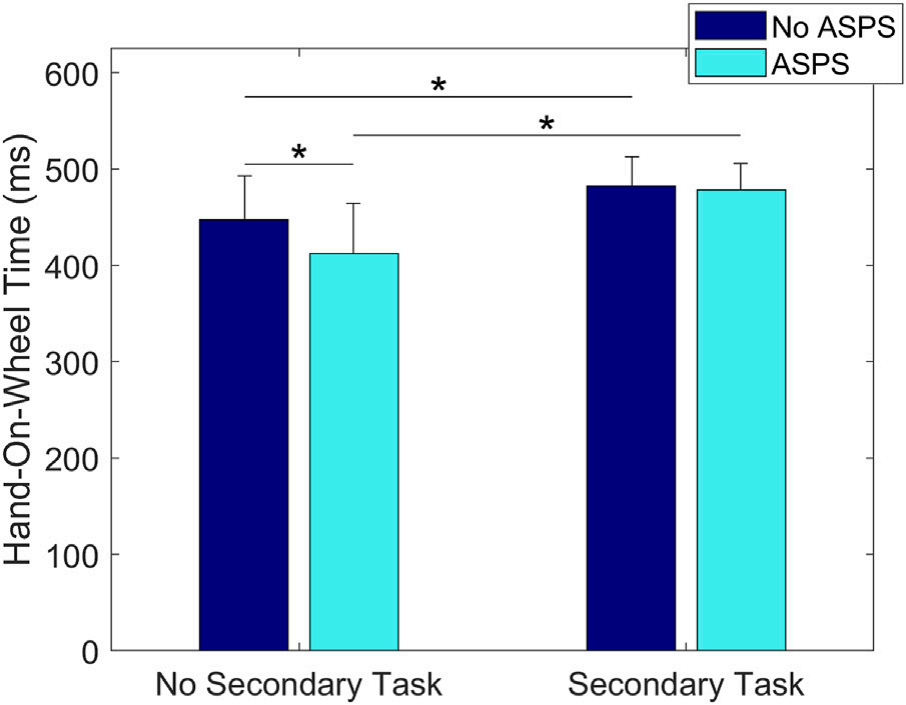
Group means ± SD for Hand-On-Wheel Time with and without the ASPS in conditions without and with the Secondary Task.

Results showed a statistically significant main effect of ASPS on Hand-Lift-Off Time, where the time was shorter with the ASPS (169 ± 55 ms) than without (194 ± 46 ms) (*p* = 0.01, Figure 5). A statistically significant interaction effect of ASPS and Age was found on Hand-On-Wheel Time, showing that adult drivers’ Hand-On-Wheel Time was shorter with the ASPS (435 ± 54 ms) than without (470 ± 33 ms) (*p* = 0.01, Figure 6), but there was no significant effect of ASPS on the teenage drivers (*p* > 0.98). A statistically significant interaction effect of ASPS and Secondary Task was also found on Hand-On-Wheel Time, showing that when there was no Secondary Task, Hand-On-Wheel Time was shorter with the ASPS (412 ± 52 ms) than without (447 ± 45 ms) (*p* < 0.001, Figure 7). With the Secondary Task, drivers showed a slower Hand-On-Wheel Time (*p* < 0.001) regardless the presence of the ASPS (478 ± 27 ms vs. 412 ± 52 ms with ASPS; 482 ± 30 ms vs. 447 ± 45 ms without ASPS, Figure 7).

#### 3.1.2 Sex

**FIGURE 8:**
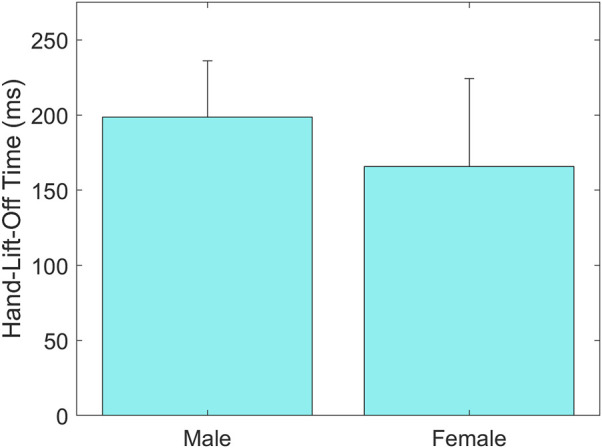
Group means ± SD for Hand-Lift-Off Time for male and female drivers.

A statistically significant main effect of Sex was found on Hand-Lift-Off Time (*p* = 0.009), showing that female drivers lift their hands towards the wheel quicker than male drivers (females: 166 ± 58 ms vs. males: 199 ± 36 ms, Figure 8).

**FIGURE 9:**
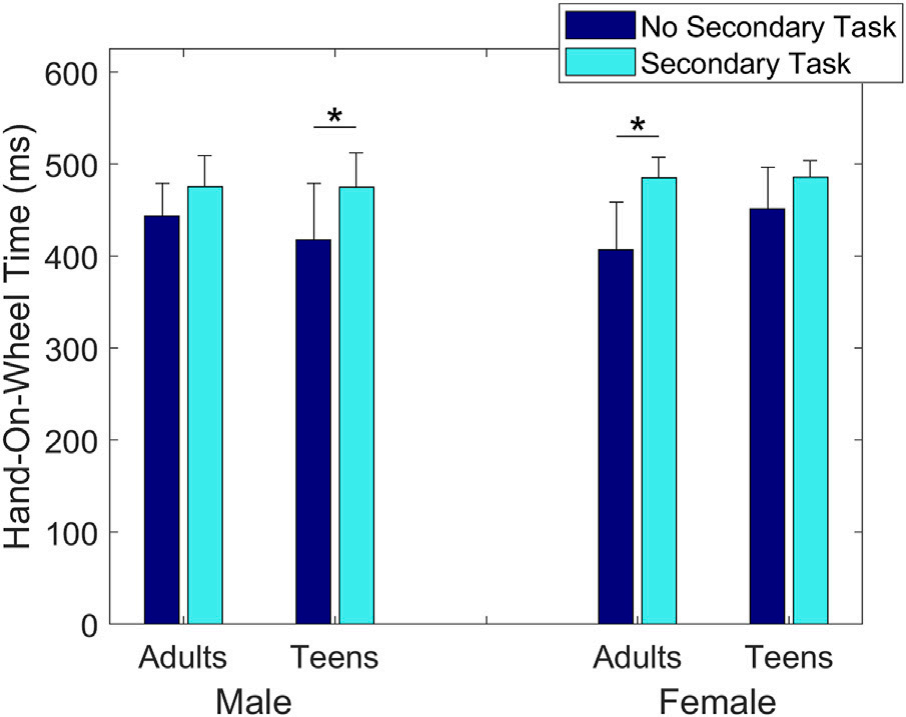
Group means ± SD for Hand-On-Wheel Time for each sex and age group of drivers without and with the Secondary Task.

A statistically significant three-way interaction effect of Sex, Age and Secondary Task was found on Hand-On-Wheel Time, showing that male teenage subjects had a shorter Hand-On-Wheel Time without the Secondary Task (417 ± 62 ms) than with it (475 ± 58 ms) (*p* = 0.005, Figure 9); similarly, female adult subjects had a shorter Hand-On-Wheel Time without the Secondary Task (407 ± 52 ms) than with it (485 ± 22 ms) (*p* < 0.001, Figure 9). No difference was found in the male adult subjects (*p* = 0.29) or in the female teenage subjects (*p* = 0.21).

#### 3.1.3 Secondary task

**FIGURE 10:**
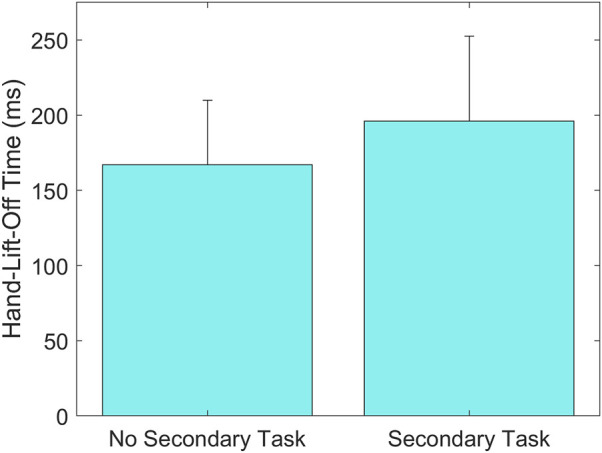
Group means ± SD for Hand-Lift-Off Time without and with the Secondary Task.

A statistically significant main effect of Secondary Task was found on Hand-Lift-Off Time (*p* < 0.001), showing that drivers lifted their hands towards the wheel quicker in trials without the Secondary Task (184 ± 43 ms) than in trials with the Secondary Task (196 ± 56 ms) (Figure 10).

Corrective time did not show any statistically significant differences across all factors (ASPS, Sex, Age, and Secondary Task), *p* > 0.09.

### 3.2 Kinematics

**FIGURE 11:**
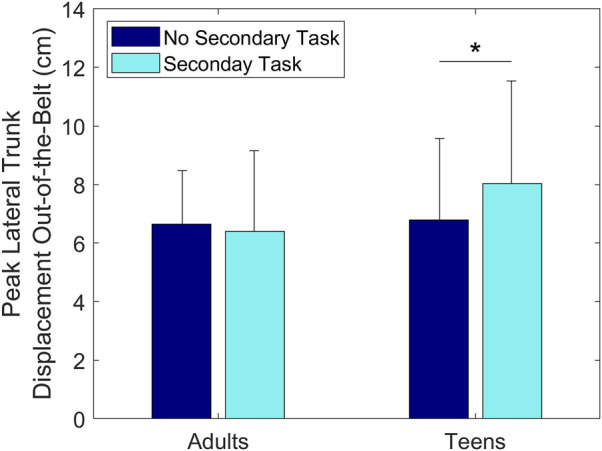
Group means ± SD for Peak Lateral Trunk Displacement Out-of-the-Belt for adult and teenage drivers without and with the Secondary Task.

A statistically significant interaction effect of Age and Secondary Task was found on Peak Lateral Trunk Displacement Out-of-the-Belt, showing that this displacement was greater for teenage subjects in trials with the Secondary Task (8.0 ± 3.4 cm) than in trials without the Secondary Task (6.8 ± 2.7 cm) (*p* < 0.02, Figure 11).

An interaction effect of Sex and Secondary Task was found on Peak Lateral Trunk Displacement Into-the-Belt, although the level of significance was weak (*p* = 0.048) and *post hoc* comparisons did not show any statistical significance differences (*p* > 0.2).

**FIGURE 12:**
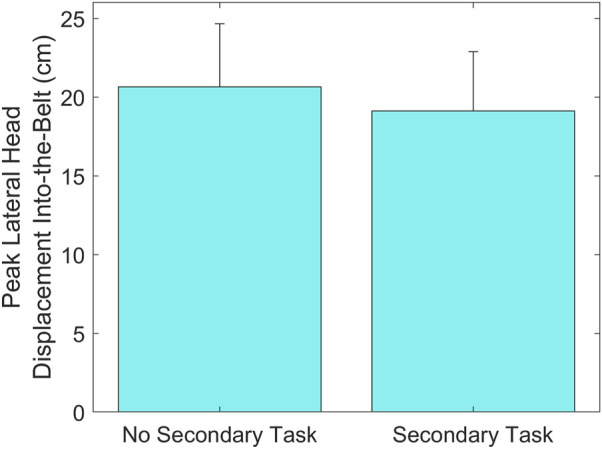
Group means ± SD for Peak Lateral Head Displacement Into-the-Belt without and with the Secondary Task.

A statistically significant main effect of Secondary Task was found on Peak Lateral Head Displacement Into-the-Belt (*p* = 0.004), showing that this displacement was greater in trials without the Secondary Task (20.6 ± 4.0 cm) than in trials with the Secondary Task (19.1 ± 3.7 cm) (Figure 12).

Peak Lateral Head Displacement Out-of-the-Belt did not show any statistically significant differences across all factors (ASPS, Sex, Age, and Secondary Task), *p* > 0.1.

### 3.3 Takeover strategies

Results showed that adult subjects used two hands to touch the steering wheel in 70.5% of trials (79 out of 112), while teenage subjects use two hands in only 50.0% of trials (56 out of 112). Female adults used two hands to touch the steering wheel in 75.0% of trials (42 out of 56), the most of all sex and age groups. It was also found that in trials without the Secondary Task (both without and with ASPS), subjects used two hands to touch the steering wheel in 87.5%–89.3% of trials (49/56 and 50/56), while in trials with the Secondary Task (both without and with the ASPS), subjects used two hands to touch the steering wheel in only 30.4%–33.9% of trials (17 and 19 out of 56).

After finding these differences in number of hands used to touch the steering wheel, a secondary analysis was conducted to understand if this wheel-touch strategy affected the time it took to place the hand/hands on the steering wheel, i.e., Hand-On-Wheel Time. A statistically significant difference was found, showing that in trials in which the subject use only one hand to touch the steering wheel (89 out of 224), the Hand-On-Wheel Time was longer (475 ± 42 ms) than in trials in which the subject used both hands (135 out of 224 trials; 442 ± 60 ms; *p* < 0.001).

Results showed that overall, subjects in all groups used the same hand to touch the steering wheel as the hand that lifted first (i.e., “matching”) in approximately 50% of trials. However, it was found that in trials with the Secondary Task (both without and with the ASPS), subjects only used matching hands in 37.0%–38.9% of trials (20/54 and 21/54).

For trials in which the subject used only one hand to touch the steering wheel, it was found that the hand that lifted first and the hand used to touch the steering wheel were matching in 43.5% of trials (37 out of 85), whereas in 56.5% of trials (48 out of 85) the hand used was not matching.

## 4 Discussion

The aim of this study was to understand if an ASPS decreases takeover reaction time for drivers in a critical autonomous driving scenario both with and without a secondary task, and to examine if sex and age influence the effectiveness of ASPS.

The results showed that overall, drivers lifted their hands from their lap on average 25 ms faster when exposed to ASPS, regardless of whether they were engaged in a Secondary Task or not. Without the Secondary Task, drivers also touched the steering wheel on average 35 ms faster when exposed to ASPS, but no influence of ASPS was seen in the time to complete the steering action. These results together suggest that the ASPS could be particularly effective early in the takeover action, specifically in the orientation stage when a drivers’ motor response first begins (i.e., the hands lift off). However, in real world scenarios, the orientation stage could vary based on occupants’ position and muscular activation during a secondary task. Future studies exploring a variety of initial postures are needed to understand the full effectiveness of the ASPS during the orientation phase. In the initialization stage of the takeover action (i.e., hands on the steering wheel), the ASPS is effective only in conditions where the driver is prepared to react and not engaged in a secondary task. By the latest stage of the takeover action, action-execution (i.e., driver completed steering), the ASPS did not show any effect. This is in line with previous research that found that the ASPS can be effective in accelerating simple motor actions, such as ankle flexion (Sutter et al., 2016). In this study, we examined a multi-segment motion that began with a simple motor response (i.e., lifting the hands off the lap) and ended with a more complicated motion (i.e., turning a steering wheel during a sled perturbation). These results show the potential of the ASPS to accelerate the orientation and initialization of the takeover action, but further research may be necessary to explore other factors that influence the effectiveness of the ASPS. For instance, although no effect of ASPS was seen on the head and trunk motion in the current study, a preliminary analysis on only the male drivers found ASPS to reduce Peak Lateral Trunk Displacement Out-of-the-Belt (Graci et al., 2019). In addition, although the average decrease in Hand-Lift-Off Time with the ASPS was only 25 ms, for some subjects this difference was greater than 100 ms, up to 228 ms. Some of these larger differences in reaction times may be enough to allow the driver to begin a crash avoidance maneuver and/or reduce their head or trunk excursion so that a more optimal position within the seatbelt is achieved before a potential crash.

Our findings suggest that the ASPS is more effective for adult drivers than for teenage drivers, as the ASPS accelerated both Hand-Lift-Off Time and Hand-On-Wheel Time in adult drivers, while it only accelerated Hand-Lift-Off Time in teenage drivers. Adult drivers were also found to reach for the steering wheel with two hands more frequently than teenage subjects, which was shown to be faster on average than reaching for the steering wheel with only one hand. It is possible that in this study, using one hand to reach for the steering wheel was slower than using two hands due in part to the fact that in about half of trials, the first hand to lift was not the same as the first hand to touch the steering wheel. Thus, in teenage subjects, who used one hand to touch the steering wheel more frequently than adults, using one hand to touch the steering wheel may have minimized the earlier positive effect of ASPS on Hand-Lift-Off. These findings are in line with previous investigations that found that teenagers engage in more risky driving behaviors (McDonald et al., 2019).

Furthermore, the results showed that female drivers lifted their hands faster than male drivers. Previous literature on simple reaction-time tasks (e.g., pressing a button) show that males are on average faster than females (Der and Deary, 2009). A previous driving simulator study on takeover found that males crash less than females (Loeb et al., 2019), which seem to be in contrast with this finding. However, studies in perception of risk in driving show females to perceive greater risk than males (Rhodes and Pivik, 2011), which may lead to a greater sense of urgency and faster reaction times in more naturalistic and critical scenarios, such as the physical sled perturbation used in this study. For instance, in the 100 Car Naturalistic Study (Montgomery et al., 2014), it was shown that women tended to underestimate the time to collision to a greater degree than men. Female drivers in the current study may have demonstrated a similar earlier recognition of collision risk than male drivers, causing their initial reaction to the sled perturbation to occur quicker than that of the male drivers. Our findings seem to indicate that female drivers assume a more cautious driving behavior during critical pre-crash scenarios.

Another difference in sex and age was seen in the Hand-On-Wheel Time with and without the Secondary Task. Without the Secondary Task, male teenagers and female adults both showed a statistically significant decrease in Hand-On-Wheel Time, while male adults and female teenagers did not. This similar result in groups that are distinct in both sex and age demonstrates the importance of taking into account both sex and age when designing warning systems. The decrease in reaction time without the phone for male teenagers may seem in contrast with previous studies on driving behavior, showing young male drivers to react later than other drivers (e.g., Montgomery et al., 2014). However, studies on simple reaction time tasks show the shortest reaction times in males 18–20 years old (Der and Deary, 2009). This suggests that the male teenagers would have a physiological advantage when the Secondary Task is not present. Recent efforts aimed at automatically silencing cell phone notifications while driving (e.g., Apple’s Driving Focus or Google Assistant’s Driving Mode) may therefore prove additionally beneficial to this population of drivers.

Overall, the Secondary Task was detrimental to drivers’ reaction times, increasing their Hand-Lift-Off Time and minimizing the effectiveness of the ASPS on Hand-On-Wheel Time. The ASPS reduced the time to touch the steering wheel for all subjects, but only when they were not engaged in the Secondary Task. When subjects were texting, the ASPS had no effect on this measure, suggesting that the ASPS is more effective for drivers who are prepared to react, as previously stated. The ASPS may therefore be effective in accelerating reaction times if used after a traditional warning, such as FCW, which prepares the driver. Moreover, the Secondary Task increased the time to reach the steering wheel both in conditions with ASPS and without ASPS. These findings are in agreement with previous literature showing that engaging in a Secondary Task while driving is detrimental to the driver’s preparedness to execute a corrective maneuver (Hancock et al., 2003; Eriksson and Stanton, 2017). Moreover, the results suggest that using one hand to reach for the steering wheel, which occurred more frequently when the drivers were engaged in the Secondary Task, slowed reaction times. Additionally, for the teenage group, the presence of the Secondary Task also resulted in greater trunk displacement out of the seatbelt, resulting in a more disadvantageous position within the seatbelt. However, across all subjects, the presence of the Secondary Task resulted in decreased head displacement into the seatbelt. This effect may be due to subjects’ gaze being on the phone during the first sled motion, which may influence their head orientation and thus explain why the decreased displacement into the seatbelt is only present in the head and not the trunk.

This study has some limitations. A small age range was used for the teenage population (16.9–17.8 years old) to include only relatively inexperienced teenage drivers and avoid spurious results due to variability in driving experience. The differences in reaction times observed between teenage and adult drivers in this study are likely due to the effect of age, rather than differences in driving experience, since both groups of drivers included in the study were inexperienced with SAE Level 3 automation (SAE J3016, 2018) as well as the specific task they were instructed to perform in this study. Moreover, previous studies found no significant effect of manual driving experience on takeover time (Chen et al., 2021). Another limitation was that participants were not given instructions on how to grab the steering wheel (e.g., with one or two hands) in order to capture the natural behavior of the drivers during a critical autonomous scenario. The lack of instruction may have impacted reaction times since a greater variability in use of one or two hands was seen in the teenage subjects than in the adult subjects, which may have minimized the influence of the ASPS in the teenage drivers. A final limitation was that this was a laboratory study and not a naturalistic environment so participants may have been prepared to react. However, several measures to reduce anticipation were employed, including a randomized start time and catch trials with no sled motion.

This study demonstrated the potential benefit of a novel startle-based warning on driver reaction times during the initiation of a corrective maneuver in a critical autonomous driving scenario. This study also highlighted the potential benefit of tailoring autonomous vehicle warnings to the age and sex of the driver. Further research is needed to understand how to fine-tune the ASPS design to potentiate its efficacy across all driving scenarios and occupants of different age and sex.

## Data Availability

Data available on request due to privacy/ethical restrictions.
