# Supplementary material for: The effect of a startle-based warning, age, sex, and secondary task on takeover actions in critical autonomous driving scenarios
Source: Front Bioeng Biotechnol. 2023 Mar 27;11:1147606. doi: 10.3389/fbioe.2023.1147606 (PMC10083268; doi:10.3389/fbioe.2023.1147606)
Supplement: Supplementary file 1 [file Table1.DOCX]

Supplementary Material

The Effect of a Startle-Based Warning, Age, Sex, and Secondary Task on Takeover Actions in Critical Autonomous Driving Scenarios

M. Griffith^1^, R. Akkem^1,2^, J. Maheshwari^1^, T. Seacrist^1^, K.B. Arbogast^1,3^, V. Graci^1,2*^

^1^Center for Injury Research and Prevention, Children’s Hospital of Philadelphia, Philadelphia, PA, USA

^2^School of Biomedical Engineering, Science, and Health Systems, Drexel University, Philadelphia, PA, USA

^3^Perelman School of Medicine, University of Pennsylvania, Philadelphia, PA, USA

*** Correspondence:**Valentina Graci, PhD
graciv@chop.edu

# Supplementary Figures

**
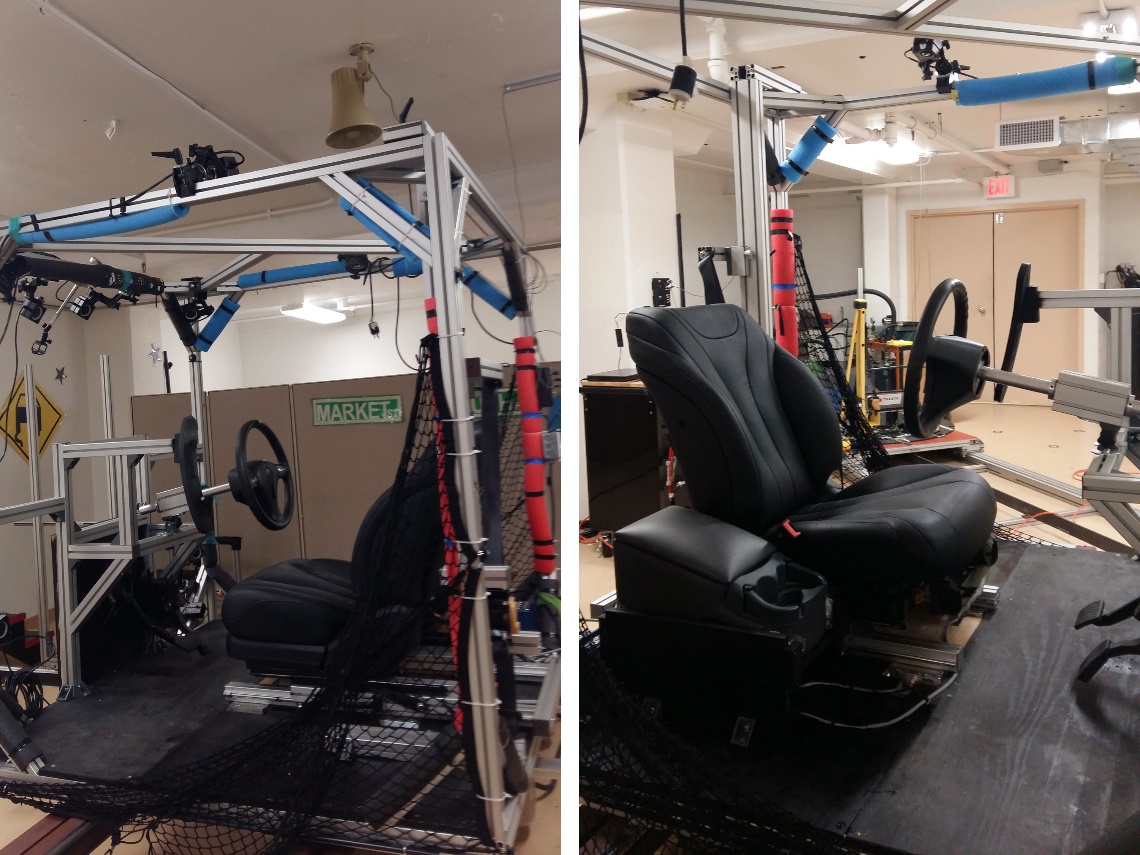
**

**Supplementary Figure 1.** Driver compartment of the sled apparatus used for experimental testing without a subject seated in it, as viewed from two sides (A, B).
